# Successful Management of Vaginal Cuff Dehiscence With Peritonitis by Elective Laparoscopic-Assisted Repair: A Case Report

**DOI:** 10.7759/cureus.94058

**Published:** 2025-10-07

**Authors:** Sho Kudo, Hideaki Tsuyoshi, Akiko Shinagawa, Makoto Orisaka, Yoshio Yoshida

**Affiliations:** 1 Obstetrics and Gynecology, University of Fukui, Eiheiji-cho, JPN

**Keywords:** bowel evisceration, laparoscopic surgery, total laparoscopic hysterectomy, transvaginal repair, vaginal cuff dehiscence

## Abstract

Vaginal cuff dehiscence (VCD) is a rare complication of total laparoscopic hysterectomy (TLH); the incidence rate has been reported to be 1.27%. However, a definitive treatment strategy has not yet been established. To our knowledge, we report the first case of VCD that was successfully managed with elective laparoscopic-assisted vaginal repair following initial conservative antibiotic therapy.

A 39-year-old woman presented with abdominal pain four months after TLH. She was diagnosed with a 2-cm VCD accompanied by abscess leakage and peritonitis. After confirming the absence of bowel evisceration, she was initially treated with intravenous antibiotics for seven days. After achieving infection control, a laparoscopic-assisted transvaginal repair was successfully carried out. The patient’s postoperative course was uneventful, and no recurrence was observed at the 3-month follow-up.

For VCD complicated by peritonitis but without bowel evisceration, performing elective repair after controlling the infection with antibiotics can be considered a safe and effective management strategy.

## Introduction

Vaginal cuff dehiscence (VCD) is a relatively rare, specifically 1.27%, complication following total laparoscopic hysterectomy (TLH) [1]. It has been reported that most cases occur within three months of surgery (median: 8.4 weeks), often triggered by sexual intercourse or straining [2,3].

VCD is often discovered due to genital bleeding or intestinal prolapse, which may lead to sepsis or intestinal necrosis and is associated with a high mortality rate, about 6% [3]. Postoperative infection, advanced age, chronic cough, early sexual intercourse, and other factors that impair wound healing have been reported as risk factors [4]. Therefore, appropriate diagnosis and management are essential, although no standardized management strategy has been established for VCD [5-15]. In particular, although it has been pointed out that the presence of infection can cause suture failure [16], to our knowledge, there have been no previous reports of VCD cases undergoing elective repair after antibiotic treatment, and the effectiveness of this approach has not yet been verified. In cases involving intestinal prolapse, emergency surgery is required to preserve the intestine. In all previous reports, emergency surgery was performed. However, whether elective surgery is a feasible option in cases without intestinal prolapse remains uncertain. Herein, we report the first case in which laparoscopic-assisted elective surgery following conservative antibiotic treatment was effective for VCD after TLH.

## Case presentation

A 39-year-old woman (gravidity 3, parity 2, 2 vaginal deliveries and 1 artificial abortion). She had no relevant medical history and was taking a low-dose oral contraceptive pill for acne treatment. Conization was performed because a malignant tumor was detected on cervical biopsy, and postoperative pathological examination revealed cervical intraepithelial adenocarcinoma. Laparoscopic total hysterectomy (TLH) was performed as an additional treatment. During surgery, cord-like adhesions were found in the ileocecal area and abdominal wall and were dissected; however, no adhesions were observed elsewhere. No gross abnormalities were observed in the uterus or adnexa.

**Figure 1:**
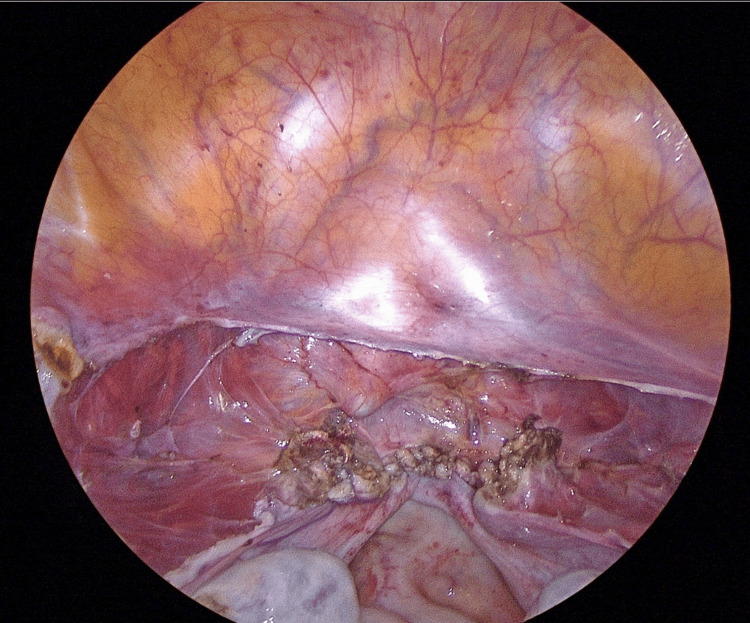
Vaginal cuff (first operation)

The vaginal cuff was cut using an ultrasonic coagulation device by elevating the anterior and posterior vaginal vaults with an intravaginal tube. The vaginal cuff was sutured in two continuous layers (only the vaginal wall is sutured layer by layer, not including the peritoneum) using a 2-0 multifilament synthetic absorbable suture (Figure 1).

**Figure 2:**
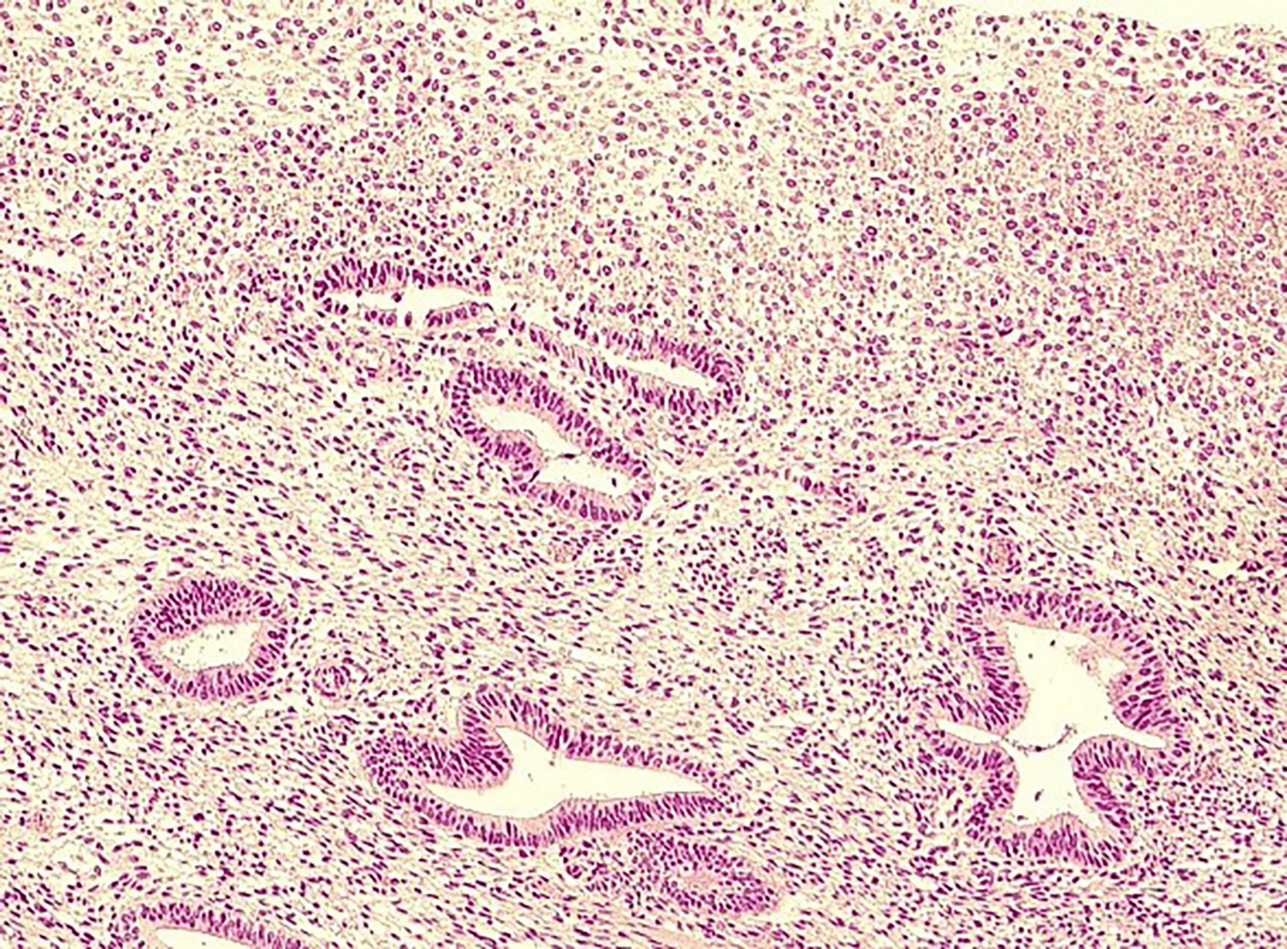
Histopathological findings of hysterectomy specimens

After suturing, a local hemostatic agent and an adhesion barrier were applied to the vaginal cuff. The operation lasted 2 hours and 48 minutes, and blood loss was minimal and not quantifiable. The postoperative course was uneventful, and the patient was discharged on the fourth postoperative day. No abnormalities were observed in the vaginal cuff prior to discharge. The final diagnosis based on the resected specimen was cervical intraepithelial adenocarcinoma and high-grade cervical dysplasia. As the resection margin was negative, the patient was followed up on an outpatient basis without additional treatment (Figure 2).

**Figure 3:**
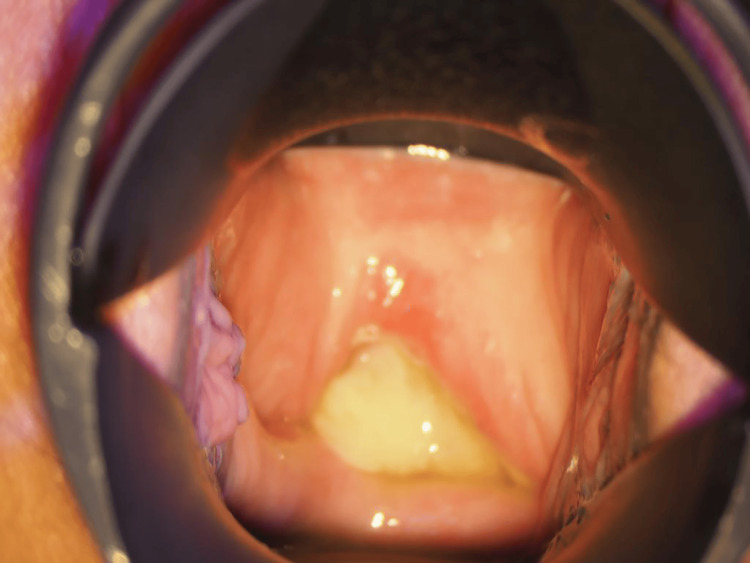
Vaginal stump dehiscence findings

The patient’s postoperative course was initially uneventful. However, she suffered from chronic constipation, the same as before surgery, and she removed restrictions on sexual intercourse at her own discretion. On postoperative day 115, she presented with abdominal pain. A 2-cm dehiscence of the vaginal cuff was identified, accompanied by abscess discharge, and intestinal peristalsis within the abdominal cavity was visible through the dehiscence (Figure 3).

**Figure 4:**
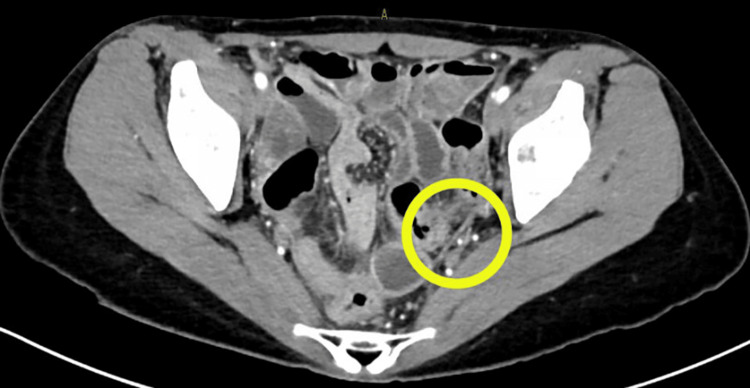
Pelvic computed tomography (CT) with contrast

Blood tests revealed a marked increase in the white blood cell count (14,500/μL) and in the C-reactive protein level (19.02 mg/dL). A computed tomography (CT) scan revealed mild thickening of the pelvic peritoneum, leading to a diagnosis of peritonitis (Figure 4).

**Figure 5:**
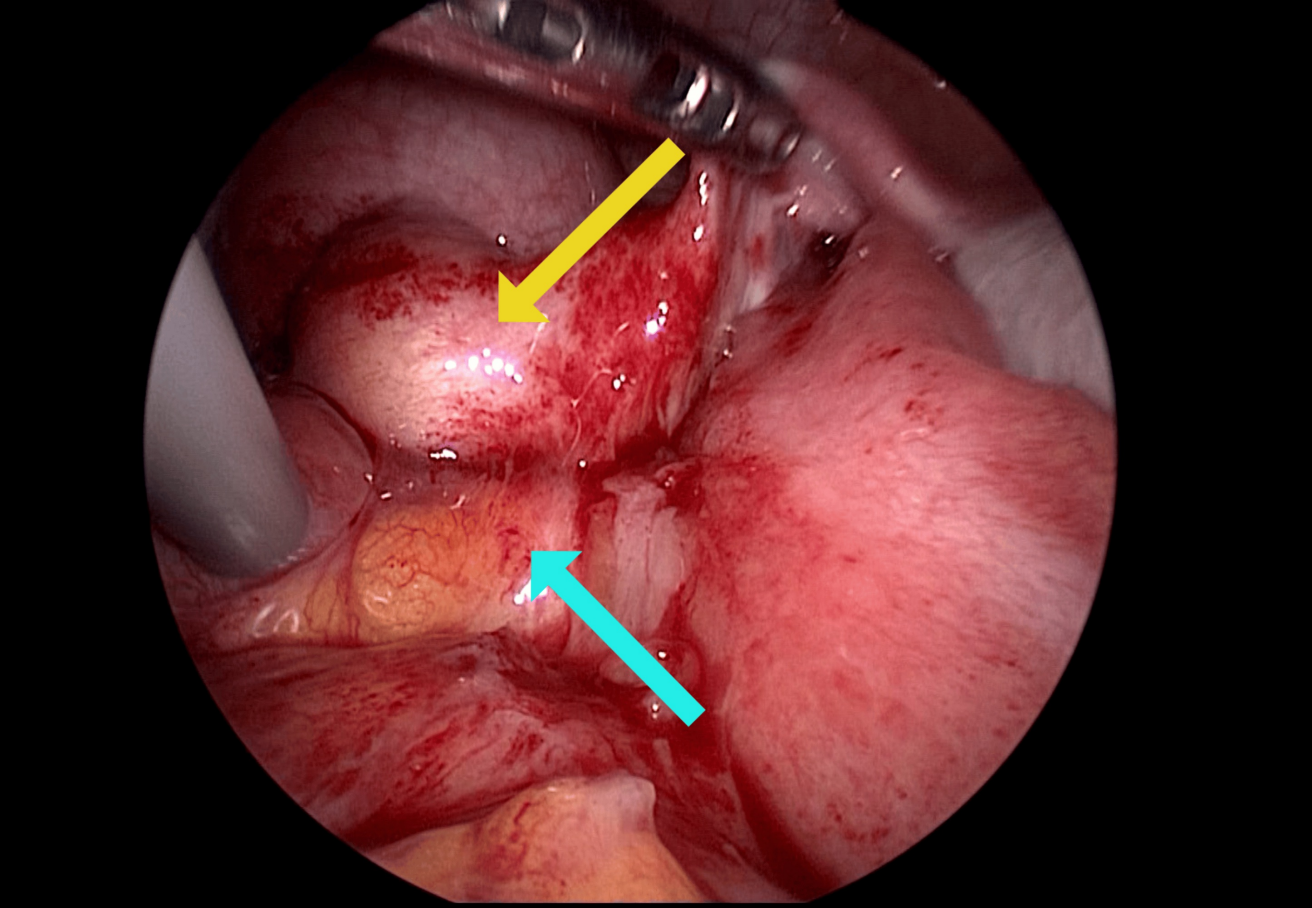
Adhesion at the vaginal cuff

The patient was diagnosed with VCD following TLH. Initial management consisted of antibiotic therapy, followed by resuturing after infection control had been achieved. Cefmetazole (2 g/day) was administered for 7 days starting from the day of admission empirically, and *Streptococcus pyogenes* was identified in a vaginal culture. After confirming improvement in laboratory findings and resolution of peritoneal irritation, a laparoscopic-assisted transvaginal resuturing was performed on hospital day 8. During the operation, the vaginal stump was overlaid by the small intestine, and adhesions were observed between a portion of the vaginal cuff (Yellow arrow) and the small intestine (Light blue arrow) (Figure 5).

**Figure 6:**
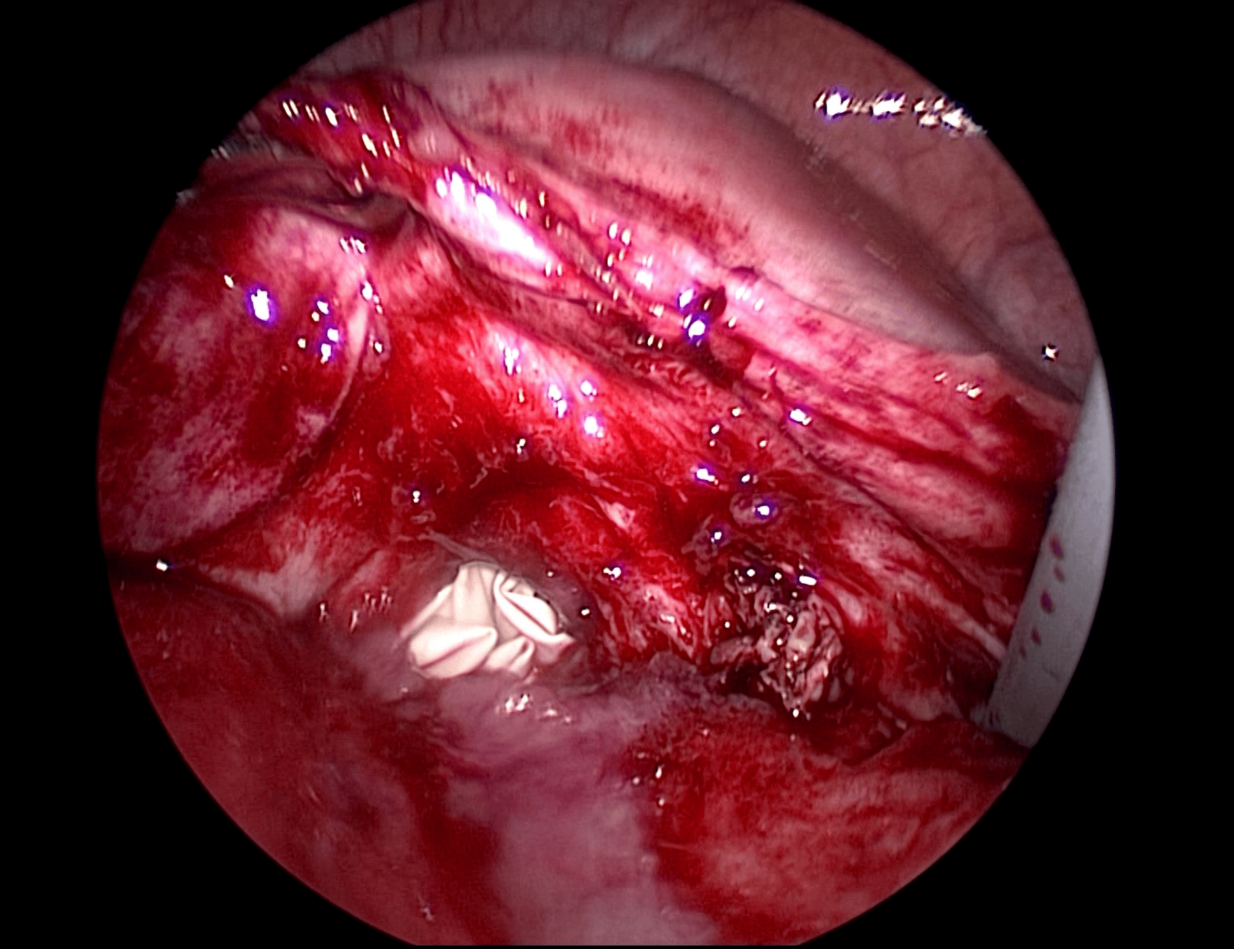
After release of adhesions

A surgical glove filled with gauze was placed inside the vagina to prevent leakage of pneumoperitoneum gas from the vaginal side, and the surrounding tissue was dissected to expose the dehisced area (Figure 6).

**Figure 7:**
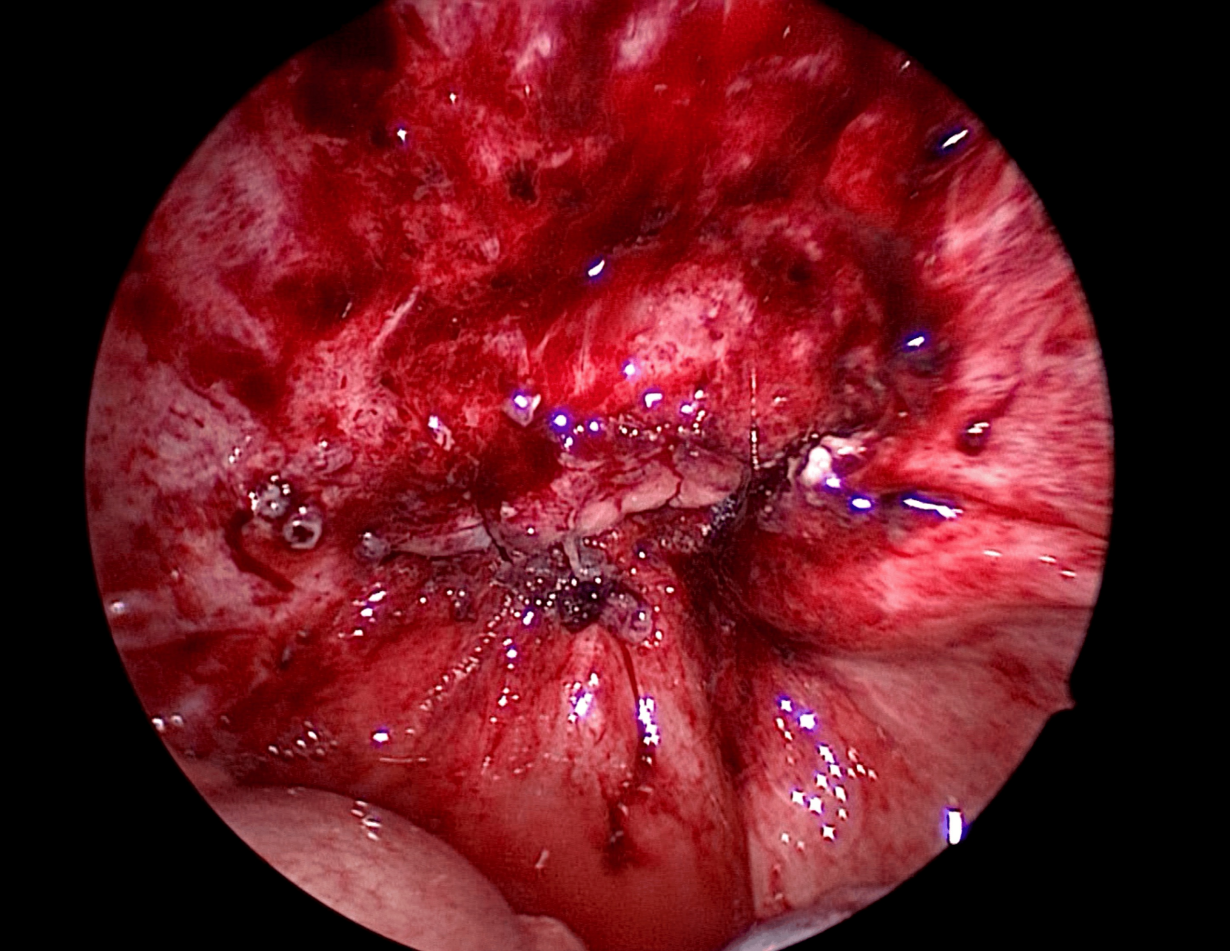
After suture of the vaginal wall

**Figure 8:**
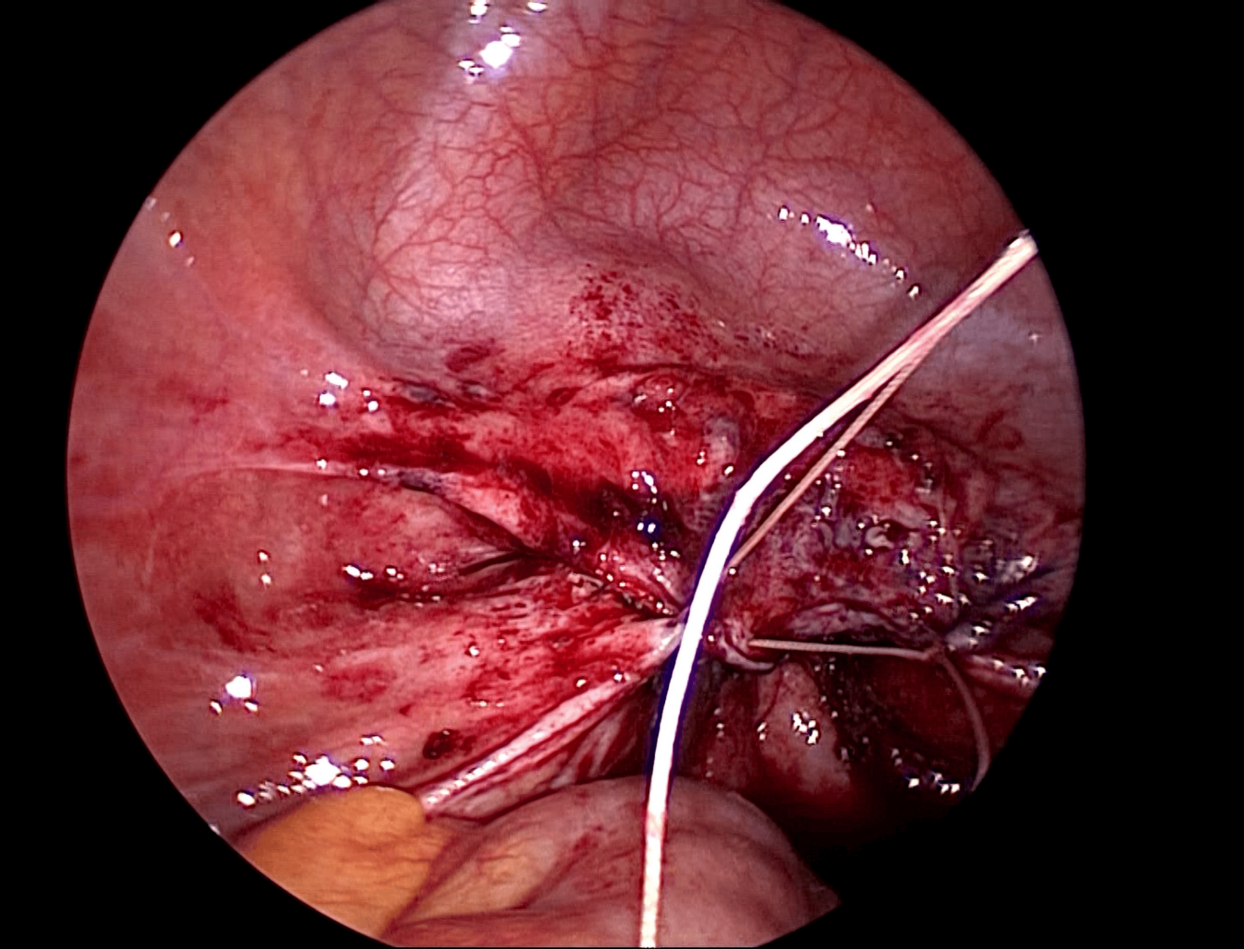
During the suture of the peritoneum

**Figure 9:**
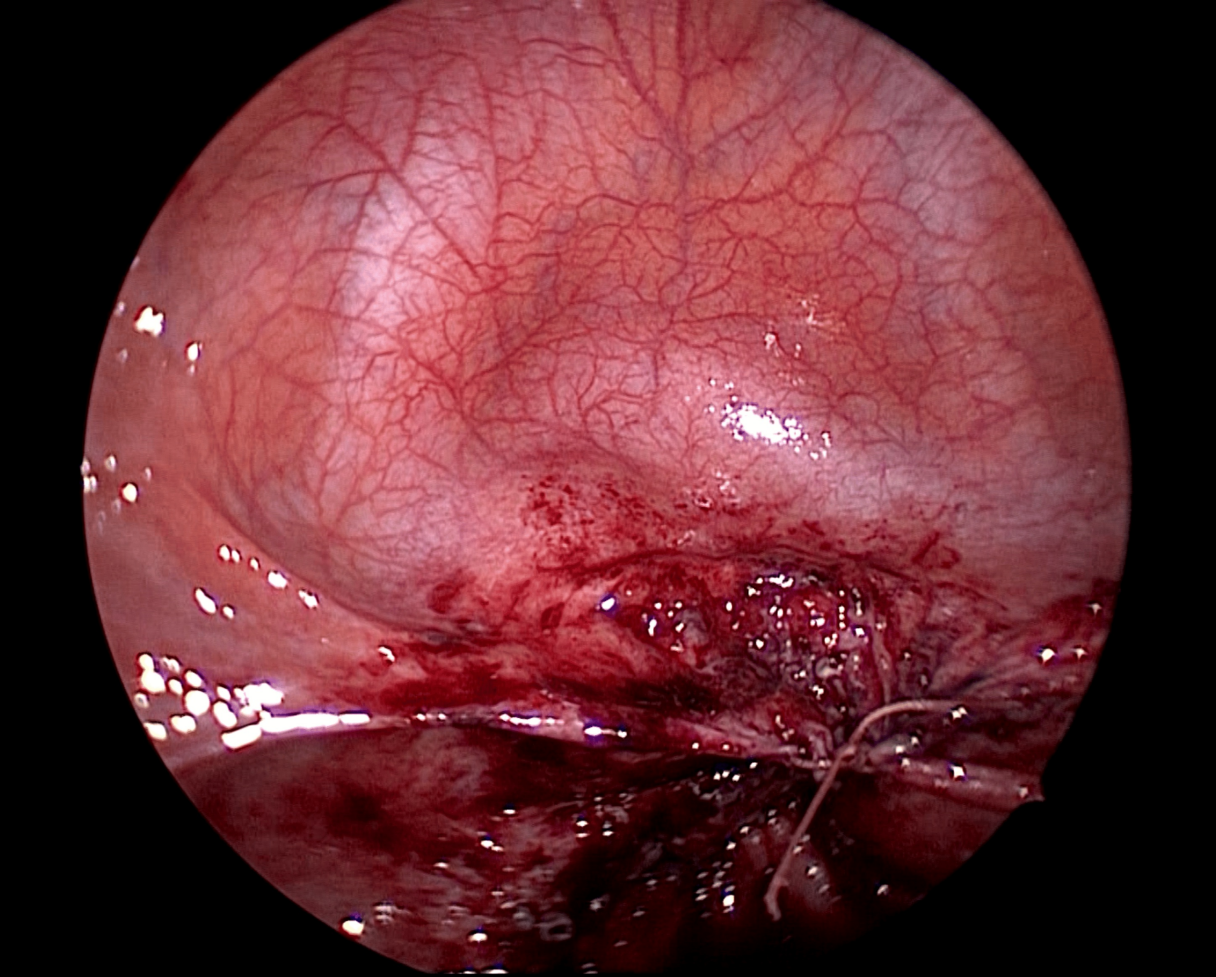
After suture of the peritoneum

The fragile tissue of the vaginal stump was debrided. After debridement, the surrounding area was irrigated copiously with normal saline. Subsequently, the wound was sutured transvaginally under laparoscopic guidance (Figures 7-9). First, the vaginal wall was sutured transvaginally while observing it from the abdominal cavity side using a laparoscope. Next, the peritoneum was sutured from the abdominal cavity side to form a two-layer suture.

**Figure 10:**
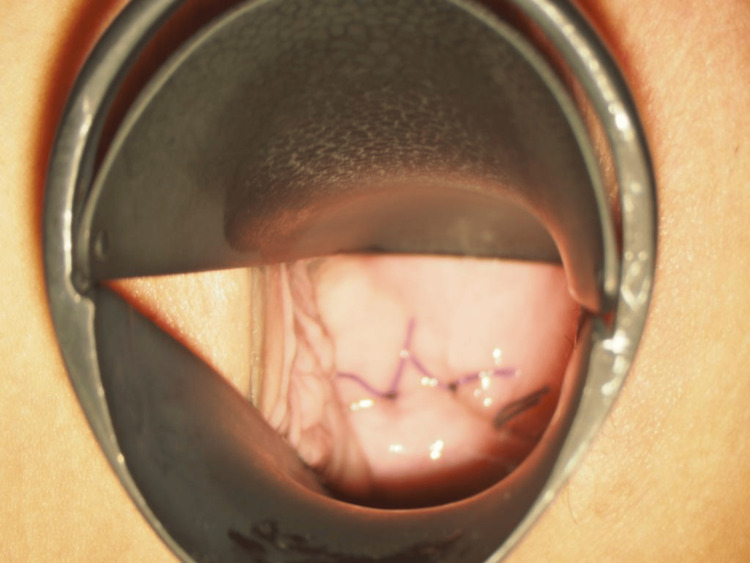
Postoperative colposcopy finding

2-0 synthetic absorbable multifilament sutures containing antibiotics were used, as these were the only antibiotic-embedded multifilament sutures available in our hospital. The patient’s postoperative course after resuturing was uneventful, and she was discharged on postoperative day 8 (Figure 10).

Three months after the second surgery, no recurrence was observed. We plan to continue to closely monitor the patient for signs of recurrence. We instructed the patient to refrain from sexual intercourse until permission was given and to use laxatives appropriately to maintain regular bowel movements.

## Discussion

To our knowledge, this is the first reported case of elective surgery for delayed VCD following TLH. The incidence of VCD after TLH has been reported to be 1.27%, which is higher than that after abdominal hysterectomy (0.02%) and vaginal hysterectomy (0.05%) [1]. The initial manifestations of VCD typically include genital bleeding and small-intestinal prolapse. If small-intestinal prolapse occurs, approximately 6% of patients die; therefore, careful management is required when VCD develops [3]. Previous studies have shown that risk factors for VCD include obesity, postoperative infection, advanced age, chronic cough, early sexual intercourse, and other factors that impair wound healing [4]. It is known that most cases are triggered by sexual intercourse or other stressful situations, such as defecation [2]. Therefore, it is important to avoid these factors as much as possible in the immediate postoperative period. Among the above risk factors, resumption of sexual intercourse two months after surgery and chronic constipation requiring frequent use of stimulant laxatives (involving daily straining) were considered relevant in this case. Considering that the average time to VCD after TLH was 8.4 weeks, resumption of sexual intercourse 2 months after surgery was clearly premature. Regarding bowel movements, this patient was barely able to have a bowel movement by using stimulant laxatives every day, and it is not difficult to imagine that she was under severe strain every day. As a supplement, the median time to onset of VCD after TLH has been reported as 8.4 weeks, earlier than that after abdominal hysterectomy (112 weeks). This may be because laparoscopic surgery is less invasive, allowing earlier recovery of activities of daily living and earlier resumption of sexual intercourse [3].

There have also been several reports regarding the relationship between surgical techniques and VCD during TLH. VCD is more common after TLH than after total abdominal hysterectomy because of the use of energy devices to incise the vaginal cuff and differences in suture strength [1]. It has been reported that double-layer vaginal cuff closure is associated with significantly fewer complications, including VCD, than single-layer closure [17]. Furthermore, monofilament sutures have been reported to be preferable to multifilament sutures [18], and barbed sutures are associated with a lower risk of VCD [19]. From the perspective of preventing VCD, it is advisable to consider these technical points when performing surgery. In particular, barbed sutures may be useful in cases with a high risk of dehiscence.

There have been previous reports describing various approaches for the treatment of VCD. Reports of VCD after TLH are summarized in Table 1.

**Table 1:** Previous reported cases of vaginal cuff dehiscence after TLH N/A: not available; TLH: total laparoscopic hysterectomy

Author/Journal [Citation No.]	Age	Primary Disease	Suturing Method (Single or Double Layer)	Energy Devices	Onset (Postoperatively)	Dehiscence Size	Intestinal Prolapse	Repair Method	Suturing Method	Restoration Date	
											Kountouri et al./Diagnostics [5]	59	Endometrioid carcinoma	N/A	N/A	6 months	N/A	Present	Laparotomy	N/A	Immediately		
Jiang et al./J Obstet Gynaecol [6]	59	Endometrioid carcinoma	N/A	N/A	5 years	N/A	Present	Laparotomy	Absorbable multifilament	Immediately		
Rathigashini et al./J Surg Case Rep [7]	47	Adenomyosis	Single layer	None	3 months	N/A	Absent	Laparoscopy	Repaired with mesh	Immediately		
Vardar and Midkiff/Radiol Case Rep [8]	40	Adenomyosis	N/A	N/A	2 months	N/A	Absent	Vaginal	N/A	Immediately		
	40	Not specified	N/A	N/A	11 weeks	2cm	Absent	Laparoscopy	N/A	Immediately		
Sendy et al./Pan Afr Med J [9]	40	Not specified	N/A	None	3 months	5cm	Present	Vaginal assisted with laparoscopy	Absorbable multifilament	Immediately		
Murray et al./Gynecol Oncol Rep [10]	68	primary peritoneal carcinoma	N/A	N/A	5 weeks	N/A	Absent	Vaginal with drain placement	Absorbable multifilament	Immediately		
											Askari et al./JSLS [11]	40	leiomyoma	N/A	Harmonic scalpel	4 months	3cm	Present	Laparoscopy	Absorbable monofilament	following day		
Newell et al. [12]	40	leiomyoma	N/A	N/A	4 months	N/A	Present	Laparoscopy	N/A	Immediately		
Houmid et al./Cureus [13]	51	leiomyoma	N/A	N/A	2 years	N/A	Present	laparotomy	Absorbable monofilament	Immediately		
Gupta et al./Cureus [14]	53	Leiomyoma	N/A	N/A	6 months	N/A	Present	Vaginal assisted with laparoscopy	Nonabsorbable monofilament	Immediately		
Robinson et al./Obstet Gynecol [15]	48	Ovarian cyst	N/A	N/A	2 months	N/A	Present	Vaginal	braided delayed absorbable multifilament	Immediately		
											Kudo	39	AIS	Double layer	Ultrasonic coagulation device	4 months	2cm	Absent	Vaginal assisted with laparoscopy	Absorbable multifilament	1week later		

Including the present case, thirteen cases have been reported. The reported patients ranged in age from 39 to 68 years, with a median age of 47 years, and the onset occurred between 11 weeks and 5 years postoperatively [5-15]. The primary indications for TLH were uterine cancer in two cases, adenomyosis in two cases, leiomyoma in four cases, benign ovarian cyst in one case, peritoneal carcinoma in one case, and unspecified in two cases. Few reports have described the surgical procedure for primary TLH, but one case involved single-layer vaginal cuff closure and another involved double-layer closure. Only two cases were documented in which the use of an energy device was documented. Of the thirteen cases, eight involved prolapse of the small intestine and six involved peritonitis (with some overlap). Repair of the dehiscence was performed by laparotomy in three cases, laparoscopy alone in four cases, a laparoscopic-assisted transvaginal approach in three cases, and a transvaginal approach alone in three cases. All thirteen cases, except the present one, were repaired immediately after diagnosis; thus, this appears to be the first reported case in which elective treatment was performed. No studies have demonstrated the superiority of open, laparoscopic, or vaginal repair; however, it has been suggested that the combined use of laparoscopy may help prevent intestinal injury [20]. In the present case, because intraperitoneal adhesions due to concomitant peritonitis were anticipated, we selected laparoscopic-assisted transvaginal suturing of the abdominal cavity rather than a vaginal operation. In addition, we chose laparoscopy instead of open surgery to minimize surgical invasiveness. During surgery, the intestine was found adherent to the stump of the dehiscence. Therefore, in cases complicated by infection, as in this case, transvaginal suturing without laparoscopic assistance should be avoided to prevent intestinal damage. When VCD is accompanied by intestinal prolapse, there is a risk of sepsis or intestinal necrosis, and early repair is required [21]. However, wound infections are known to inhibit wound healing [16]. Furthermore, it has been reported that emergency surgery has a significantly higher mortality rate than elective surgery (Odds ratio 2.91) [22]. Therefore, because there was no intestinal prolapse in this case, we prioritized antibiotic therapy for infection control. The postoperative course after resuturing was favorable, with no recurrence, suggesting that elective suturing was effective.

## Conclusions

The proportion of laparoscopic procedures among total hysterectomies is expected to continue increasing. As laparoscopic surgery becomes more widespread, the incidence of VCD is also expected to rise, highlighting the need for greater attention to surgical techniques and postoperative care to prevent VCD. Furthermore, in the event that VCD does occur, appropriate management should be individualized to the patient’s condition. Rather than performing emergency surgery in all cases of VCD, it is important to evaluate the presence of intestinal prolapse and concurrent infection and provide appropriate management accordingly. Laparoscopic-assisted transvaginal suturing after infection control may be a safe and effective strategy for managing VCD following TLH, particularly in cases complicated by peritonitis without intestinal prolapse.
